# Accounting for black carbon lowers estimates of blue carbon storage services

**DOI:** 10.1038/s41598-018-20644-2

**Published:** 2018-02-07

**Authors:** Swee Theng Chew, John B. Gallagher

**Affiliations:** Borneo Marine Research Institute, Universiti Malaysia Sabah, Jalan UMS, 88400 Kota Kinabalu Sabah, Malaysia

## Abstract

The canopies and roots of seagrass, mangrove, and saltmarsh protect a legacy of buried sedimentary organic carbon from resuspension and remineralisation. This legacy’s value, in terms of mitigating anthropogenic emissions of CO_2_, is based on total organic carbon (TOC) inventories to a depth likely to be disturbed. However, failure to subtract allochthonous recalcitrant carbon overvalues the storage service. Simply put, burial of oxidation-resistant organics formed outside of the ecosystem provides no additional protection from remineralisation. Here, we assess whether black carbon (BC), an allochthonous and recalcitrant form of organic carbon, is contributing to a significant overestimation of blue carbon stocks. To test this supposition, BC and TOC contents were measured in different types of seagrass and mangrove sediment cores across tropical and temperate regimes, with different histories of air pollution and fire together with a reanalysis of published data from a subtropical system. The results suggest current carbon stock estimates are positively biased, particularly for low-organic-content sandy seagrass environs, by 18 ± 3% (±95% confidence interval) and 43 ± 21% (±95% CI) for the temperate and tropical regions respectively. The higher BC fractions appear to originate from atmospheric deposition and substantially enrich the relatively low TOC fraction within these environs.

## Introduction

**Figure 1:**
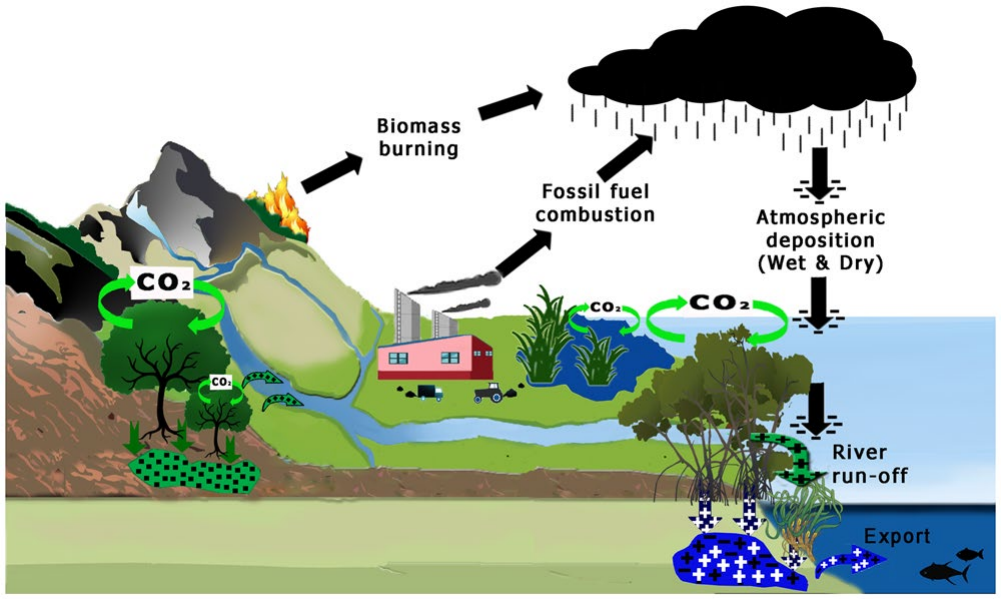
An augmented blue carbon conceptual model. The model shows the ability of saltmarsh, mangrove, and seagrass canopies to trap and store black pyrogenic carbon (BC) washed out from the catchment (green and black) and atmosphere (black), along with detritus produced by those coastal vegetated ecosystems (blue and black). BC is resistant to oxidation, and we argue that failure to remove BC and other types of allochthonous recalcitrant organic carbon, such as kerogens, from calculations of coastal vegetated habitat sedimentary carbon stocks could result in overestimated values used to estimate the mitigation service provided by these stocks in terms of anthropogenic emissions of CO_2_. Icon credit: Tracey Saxby (mangrove) and Catherine Collier (seagrass), Integration and Application Network, University of Maryland Center for Environmental Science (ian.umces.edu/imagelibrary/).

There is broad consensus that human activities are changing the global climate through anthropogenic emissions of CO_2_^1^. This realisation has highlighted the importance of conserving and enhancing existing carbon reservoirs. While terrestrial forests have received most of the attention, it is being increasingly acknowledged that blue carbon ecosystems, such as seagrass, mangrove and saltmarsh, may also play a significant role^2^. These coastal ecosystems are highly productive and have the ability to trap and deposit large quantities of both autochthonous and allochthonous organic detritus from adjacent land and coastal waters (Fig. 1). These sedimentary organic carbon stocks represent a legacy of process and preservation in mitigating further emissions of greenhouse gases. The value of these stocks is estimated based on carbon sediment content to a depth likely to be disturbed by human activities^3,4^. The importance of the legacy was highlighted by Pendleton’s group^3^. They estimated that the loss of seagrass, mangrove, and saltmarsh habitats has resulted in the release of ~0.15–1.02 Pg yr^−1^ of CO_2_ into the atmosphere, representing an uncertain but significant fraction of current global anthropogenic emissions (9.9 ± 0.9 Pg yr^−1^)^5^.

While, the large range of uncertainty in CO_2_ emissions subsequent to loss of coastal vegetated ecosystems is, in part, due to under sampling^6,7^, the question of bias with regard to blue carbon stocks, which additional sampling cannot address, has not yet been fully tested^8^. We contend that one source of bias is the implicit inclusion of allochthonous recalcitrant organic matter within sedimentary stock estimates. That is, blue carbon ecosystems are not responsible for the production of the recalcitrant carbon, and burial does not provide additional protection from remineralisation. Consequently, allochthonous recalcitrant carbon must be excluded from the sedimentary stock when estimating its value as a mitigation service for anthropogenic emissions of CO_2_^9^.

What then, are the sources of allochthonous recalcitrant carbon? The literature suggests two major sources of allochthonous recalcitrant carbon, namely, ancient kerogens and the more contemporary black carbon (BC)^10^. Kerogens are oil-yielding compounds made up of the fossilized remnants of plant materials and from less than globally ubiquitous terrestrial shale deposits^10^. Through the process of weathering, kerogens are released from those deposits and transported in soil runoff from the land into coastal waters^10^ (Fig. 1). In contrast, black carbon is formed by the incomplete combustion of both terrestrial biomass and fossil fuels; it is wide spread and transported to coastal sediments via both soil washout and atmospheric deposition (Fig. 1). Of course, assigning BC as allochthonous is contingent on the assumption that coastal vegetated ecosystems cannot burn, or at least that they do not have a fire history. Certainly, this is the case for seagrasses. For mangroves, the lack of accumulation of dry litter reduces their susceptibility to ignition, but they will burn under the correct conditions^11,12^. The exceptions to this assumption are reed and cord grass saltmarshes, which are known to burn extensively on occasion^13^. Indeed, deliberate saltmarsh fires, designed to halt adjacent mangrove encroachment, have failed to do so, perhaps a testament to the resistance of mangrove forests to ignition^14^. Without this assumption, then, thorough BC analysis would require separation of allochthonous from autochthonous BC sources, which might be done, possibly with a chemical/isotopic signal; however, such analysis is outside the scope of this initial study.

Whether or not autochthonous BC is produced within some saltmarshes, and despite the possibility of mangrove ignition at some time over the life of the deposited sediments, allochthonous black carbon within non-vegetated sediments is known to make up a significant but variable fraction of the total organic carbon (TOC) content (estimates are in the range of 50 ± 40%^15^ or 15–27% of dry mass^16^). This high variability likely reflects the extent of BC sources and their distance from the deposition site^16^. Differences in the estimates may also be due to the analytical methods chosen across studies^17^. Nonetheless, it is imprudent to estimate the extent of BC stock bias within coastal vegetated sediments from the relatively extensive, though not exhaustive^18^, coastal bare sediment datasets currently available. That is to say, factors that determine the BC fraction in each of the two sedimentary environments, vegetated and non-vegetated, are likely to be either disparate or different in extent. For example, relatively lower sedimentary BC/TOC ratios could be expected within coastal vegetated sediments because of the higher rates of autochthonous and non-refractory allochthonous detrital deposition^19^. The comparison is also only valid after rates of BC supply and labile organic carbon mineralisation rates have been normalised for temperature dependence across climatic regimes. Furthermore, higher rates of mineralisation of the more labile organic fractions when bare sediments are resuspended and deposited^20^ may exaggerate these difference. Conversely, a larger BC/TOC ratio could conceivably result from the high efficiency of BC particle trapping via atmospheric deposition by the vegetated canopy^21^. In this case, a higher BC/TOC ratio can be further exaggerated by the addition of purer forms of particulate BC deposited via aeolian transport^16^ compared to the smaller BC/TOC ratio associated with soil washout. The balance between the above two sets of opposing factors remains uncertain, and data on the subject is scarce. A far as we are aware, there are only two studies that have measured both BC and TOC content in coastal vegetated sediments. Those studies were carried out within tropical (Guanabara Bay, Brazil)^19^ and subtropical (Shantou, China)^22^ mangrove ecosystems, both located adjacent to highly urbanised environments. The tropical study reported BC/TOC ratios down five sediment cores greater than 88 cm in length; values ranged from 0.53–9.09% dry mass (median 1.53%)^19^. A reanalysis of the subtropical study’s dataset of 14 surface samples showed that the BC/TOC ratios were notably larger than those in the tropical study, that is, 2.60–13.31% dry mass (median 6.01%)^22^. Clearly, there is a need for representative measurements of BC within coastal vegetated sediments over a greater range of temperature regimes and with different BC sources.

To test the extent of black carbon bias on the estimated blue carbon stock service values, we carried out a first-level assessment using two methods of isolation, chemothermal oxidation (CTO) and concentrated nitric acid oxidation (NAO) (see Methods). The sampled habitats were located across climate regimes ranging from temperate to subtropical/tropical and had evidently different degrees of BC pollution. The Salut–Mengkabong lagoon, in Sabah, Malaysia (Supplementary Figure S1), is a tropical tide–dominated system which supports mangrove forests and seagrass meadows and is located several kilometres south of the district of Tuaran (population ~100,000). The region also sits within the penumbra of smoke haze emanating from the distant and seasonal Kalimantan peat fires^23^. We also studied the seagrass meadows of Little Swanport estuary, Tasmania, Australia (Supplementary Figure S2). This estuary is also a tide–dominated system, but it is located in a temperate rural setting with a history of local forest fires and historical aboriginal fire management practices^24^. In addition, a reanalysis of surface sediment BC and TOC data was performed across a sub-tropical wetland located within the highly polluted urban region of Shantou, China^22^. The study from which that data was obtained used a chromic acid oxidation procedure (CAO), which is thought to be equivalent with CTO^17^. While the examples do not represent a full factorial set, they still serve to illustrate a likely range of BC/TOC fractions within seagrass and mangrove sediments across the globe, and they highlight the factors behind that range.

To evaluate the methods of evaluating BC content we compared the amount of BC found in different sedimentological environs across the upper and lower sections of the lagoon/estuary. Discrepancies may be explained based on expected differences in the composition of different BC mixtures, for instance, a mix may include more labile macro chars from nearby fires in combination with more refractory finer chars and soots produced from combustion at higher temperatures (see Methods). The analysis of BC/TOC fractions is based on a series of linear regression models, the parameters of which may be used to infer either aeolian transport and/or soil washout (see Results and Discussion).

## Results and Discussion

Overall, we found that the BC content down the length of the sediment cores drawn from the tropical/sub-tropical and temperate vegetated ecosystems was greater than the amount of BC in non–vegetated coastal sediments (~0.01–0.60% dry mass)^15,25–27^. For Little Swanport and Salut–Mengkabong, the BC content determined via CTO and NAO ranged between 0.01% and 1.39% dry mass (median 0.25%, *n* = 192). For the Shantou mangrove sediments, surface BC content ranged from 0.19–1.44% dry mass (median 0.49%, *n* = 14), which is greater than the range reported down mangrove sediment cores from Guanabara Bay (0.53–9.09% dry mass, median 1.53%)^19^.

### Black carbon content

**Figure 2:**
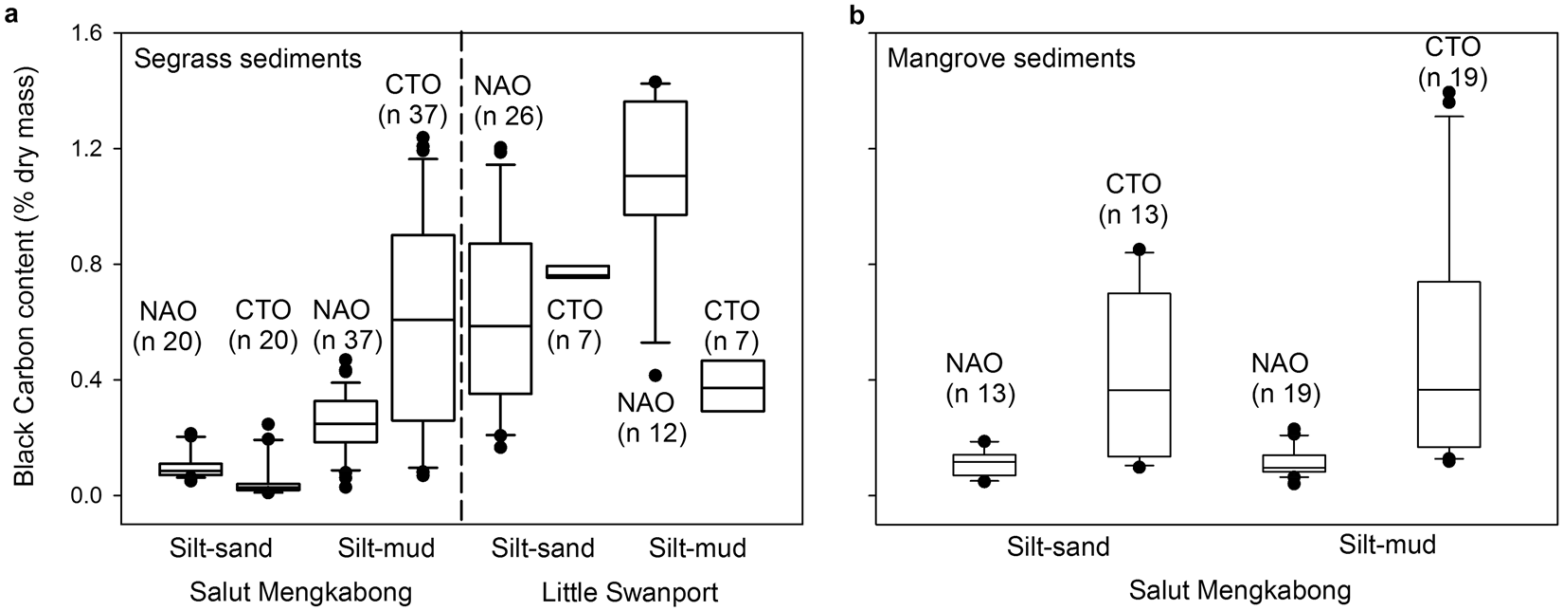
Black carbon content in seagrass and mangrove sediment cores from Salut–Mengkabong lagoon and Little Swanport estuary. (**a**) BC down seagrass sediment cores determined via chemothermal oxidation (CTO), which is thought to measure soots, and concentrated nitric acid oxidation (NAO), which is thought to measure both soots and chars. The results of the sediment analysis are separated into those for silt-sand and those for silt–mud, ostensibly within the upper and lower regions of the Salut–Mengkabong lagoon, a tropical tide-dominated system in a moderately urban environment, and Little Swanport estuary, a temperate tide-dominated estuary in a rural environment with a history of forest fires. The amount of organic carbon isolated via CTO within the upper silt–mud regions is on average greater than the amount isolated by NAO; this discrepancy suggests the existence of recalcitrant carbons other than BC (Mann-Whitney U = 14.0, *P < *0.001). (**b**) The results of sediment analysis for the mangrove forests of Salut–Mengkabong show that amounts of organic carbon isolated via CTO continue to be significantly greater than the amount isolated via NAO across the whole lagoon (for silt-sand mangrove sediments, Mann-Whitney U = 22.0, *P* < 0.004; for silt-mud sediments, Mann-Whitney U = 32.0, *P* < 0.001).

The above variability in BC contents as determined by each analytical method was confined to differences at the sampling site scale (~1 km) along the two branches of the Salut–Mengkabong lagoon (Supplementary Table S7.1–7.4). This was also covariant with sandy to muddy regions along the lagoon/estuary (Fig. 2). In other words, overall, no significant differences in BC content could be distinguished between or along individual sets of mangrove transects, and at the 10^1^ to 10^2^ m sampling station scales across the seagrass meadows. For the Little Swanport estuary, BC content varied between analytical methods rather than sampling scales (Fig. 2a and Supplementary Table S7.5, 7.6). Indeed, BC content determined via CTO was generally higher down the tropical silt-mud sediment cores of seagrasses and mangroves (Fig. 2a,b). The exception was the silt-sand cores from the tropical seagrass and mangrove areas (Fig. 2a,b), where the BC content was generally greater after NAO (Fig. 2a). The higher estimated BC content via CTO for upper seagrass and mangrove regions suggests that the ability of CTO to isolate only BC across the charcoal-soot continuum may have some constraints. There may have been charring of organic matter during combustion^28^ or other recalcitrant forms of carbon may have been present that are resistant to CTO but not to NAO. We consider the possibility of charring unlikely. Additions of sonicated microalgal slurries, which are particularly susceptible to charring, did not increase the estimates of black organic matter when added to muddy to silty sediments (see Methods). Of the possible forms of recalcitrant carbon, phytolith–occluded carbon may fit the above criteria regarding CTO and NAO in that it is thermally resistant but not chemically resistant^29,30^. Indeed, phytoliths are known to dominate biogenic silica contents in the bare sediments along the length of Malaysian river estuaries^31^ and have also been recorded down mangrove sediment cores^32^. Furthermore, the notable lack of phytoliths within Little Swanport’s seagrass sediments^33^ is consistent with the generally similar or greater levels of BC isolated by NAO over those isolated by CTO (Fig. 2a). As seagrasses have not been found to contain phytolith–occluded carbon^34,35^, its presence can also be categorised as both allochthonous and recalcitrant. However, separating mangrove phytoliths from catchment phytoliths^32^, which have no diagnostic value^35^, is a challenge that requires independent evaluation, possibly via correlations with variations in mangrove pollen.

### Black carbon fractions and supply pathways

While the above analysis was useful in assessing the methods used to estimate BC content, and determining BC hotspots, the variance in the BC/TOC fractions is best determined via a series of linear regression models across the different sampled environments of each region. We argue that linear regressions, which include an intercept, can be seen as the sum of two regressions models: first, a model that describes the proportional relationship between BC and TOC with no intercept (model’s variance is consistent with BC supplied only with soil washout down the estuary or lagoon^36^); and second, a model that describes the invariant response of BC with sedimentary TOC (i.e., regression coefficients of slope = 0 and intercept *y* > 0, respectively). The invariant response is consistent with a constant supply of BC from atmospheric deposition over the relatively small study area^36^ (several kilometres in length), regardless of whether they are soots and/or macro-chars^37^.

**Figure 3:**
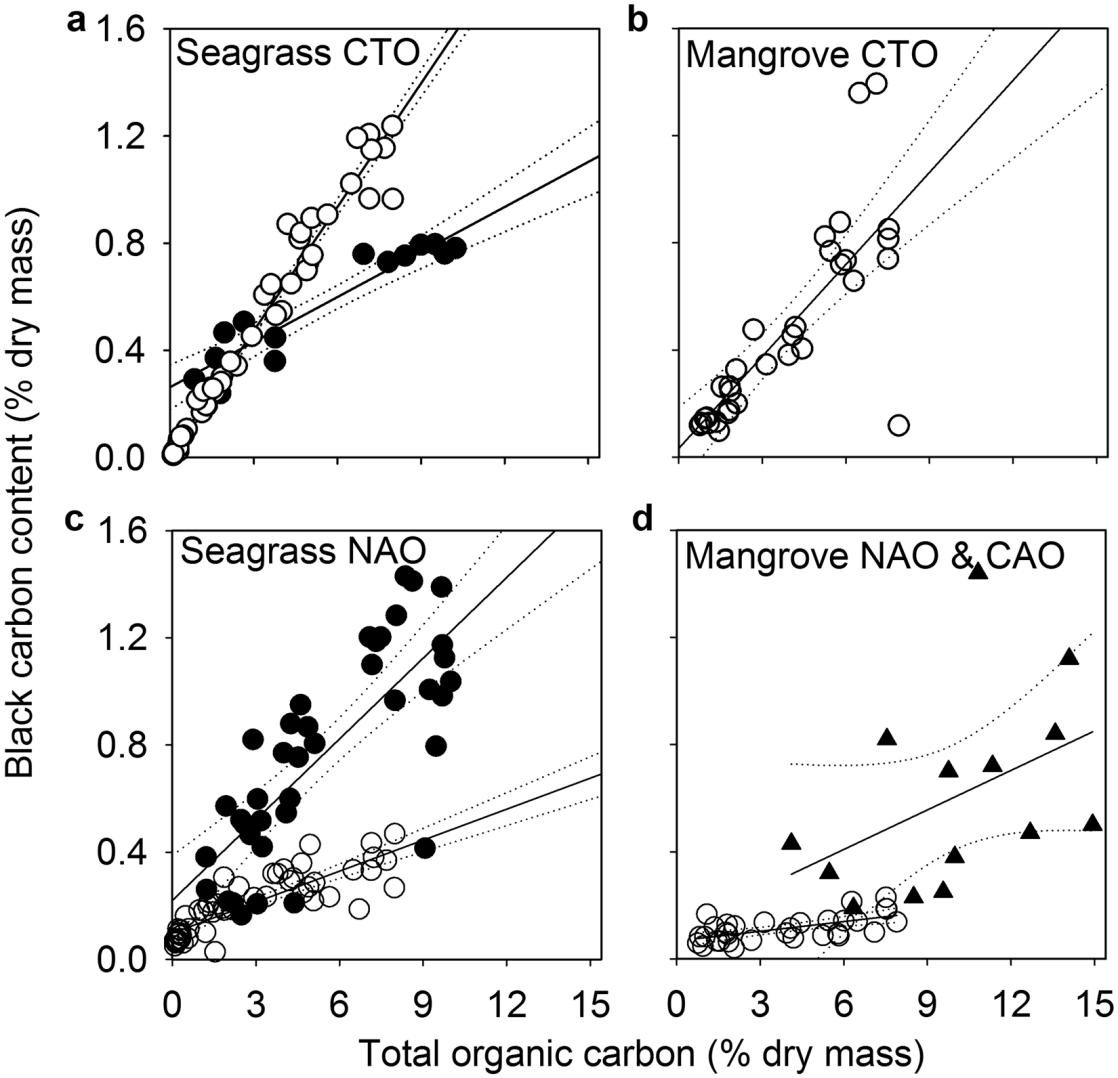
Ordinary least squares regressions for total organic carbon content against black carbon across seagrass and mangrove sediments. With the exception of Little Swanport estuary seagrass sediments, the regressions are constructed from measurements of black carbon (BC) as isolated via chemothermal oxidation (CTO), concentrated nitric acid oxidation (NAO), and dilute chromic acid oxidation (CAO) down seagrass and mangrove sediment cores along a sand-to-mud gradient of (○) the tropical lagoon Salut–Mengkabong, (●) the temperate estuary Little Swanport, and (▲) the surface sediments of the subtropical Shantou wetlands. For Little Swanport seagrass sediments, the amount of BC isolated via NAO was calculated from regressions of loss on ignition of TOC *vs* total organic matter, and black organic matter *vs* BC, constructed across the seagrass and mangrove sediments of Salut–Mengkabong (Supplementary Figures S3 and S4). The regressions represent the sum of a proportional increase in BC associated with soil carbon washout and BC supply to the sediment from aeolian transport, as measured by the intercept. The difference between the mangrove regressions (ANCOVA) from Salut–Mengkabong and the Shantou wetlands (**d**) were not statistically different (*P* = 0.15 of being the same). There was no evidence of serial correlation between the residuals; and so, the 95% confidence estimators (dotted lines) may be considered reasonable (see Supplementary Table S1 for regression equations and estimator parameters).

The parameters of the linear models of the two water bodies, that is, slope, intercept, and *R*, did not share a common response (Fig. 3). In other words, the amount of BC supplied via aeolian transport and/or from soil washout appeared to be specific to the type of ecosystem, the climatic region, and the BC source (Fig. 3). For tropical seagrass and mangrove sediments, the BC isolated by CTO represented a significant and constant fraction of the TOC, with slope coefficients of 15.4% and 11.4%, respectively (Fig. 3a,b; Supplementary Table S1 for regression equations and statistics). Furthermore, their strong correlation and near-zero intercepts are consistent with the deposition of BC and other recalcitrant forms of carbon via soil washout (Fig. 1). This is in contrast to the positive BC intercepts that are seen for both mangroves and seagrasses within temperate, tropical, and subtropical systems (Fig. 3c,d) and for BC isolated by CTO across the seagrass meadows in the temperate system (Fig. 3a). Here, the regression models suggest that BC originated from both soil washout and aeolian transport. In particular, the noticeably larger amount of BC as a fraction of TOC, as isolated by NAO, across the temperate seagrass meadows results in a greater slope coefficient in comparison to that of both tropical and subtropical seagrasses and mangrove sediments from the Shantou wetlands (Fig. 3a,b,d). A likely explanation for these differences is the greater rate of mineralisation of the more labile fractions in tropical climates, that is, higher temperatures^38^. It is also possible that the difference is exaggerated by the larger BC/TOC ratio of the temperate catchment soils attributable to local forest fires and historical aboriginal burning practices^24^. This contention is supported by a pilot study in which relatively high BC/TOC ratios isolated via CTO (14%) were found in the catchment soils adjacent to site DB6 (Supplementary Figure S2). Either way, any significant aeolian supply of BC to low-organic-content sandy sediments will likely elevate BC/TOC ratios above those of highly organic muddy sediments.

To put the above results into perspective, more typical BC/TOC ratios were calculated from the study’s linear regression models. As a precaution, chemical oxidation regressions were only used to isolate the possible inclusion of phytolith–occluded carbon (see the ‘Black carbon content’ section above). The values were calculated by first applying the global average TOC for mangrove (8%)^39^ and seagrass sediments (0.7%)^39^ to their respective regressions (Fig. 3c,d; see Supplementary Table S1b,d,f,g for equations). From the results, the matching mean BC content and the corresponding confidence intervals (CI) were used to calculate more typical BC/TOC ratios for these ecosystems and conditions. Thus, for temperate seagrass meadows located within a fire-affected region, the regression suggests a BC/0.7% TOC ratio of 43 ± 21% (±95% CI). This is in sharp contrast to the results for tropical seagrass meadows adjacent to a moderately urban areas, where the regression predicts a significantly lower average BC/0.7% TOC ratio of 18 ± 3% (±95% CI). For mangroves, the regressions predict significantly lower average BC/8% TOC ratios of 2 ± 1% (±95% CI) for the moderately polluted tropical urban area of Salut–Mengkabong and a somewhat higher ratio of 6 ± 6% (±95% CI) for the highly polluted subtropical urban area of the Shantou wetlands. The reasons for the generally lower estimates appear ostensibly because of the higher globally average mangrove sedimentary TOC content (8%) and near zero regression intercepts. It should also be noted that the overlapping variability between the two mangrove regions may be an expression of a Type II error. This contention is supported by a difference that could be discerned at a lower level of statistical power, 6 ± 4% (±80% CI), wherein the potential error may result from the larger BC variability across the highly polluted subtropical system (Fig. 3d). The higher average BC/TOC values found within the subtropical system may then truly be the result of significantly greater levels of BC pollution. Furthermore, we suggest that all the BC/TOC estimates are likely to be on the smaller side of the equation. This is primarily because the regressions were constructed within the confines of bar built lagoons and estuaries. Under these conditions, which are applicable to only a minority of seagrass meadows and mangrove forests, much of the autochthonous litter may be retained by a returning flood tide. This could conceivably result in a lower BC/TOC ratio than found in more typical open coastal ecosystems.

The BC data from the tropical and subtropical environ examples suggest that future blue carbon programs should consider adopting the notion of an allochthonous recalcitrant component of TOC. In particular, programs should measure and exclude BC content, as it is a source that is certainly allochthonous within seagrass meadows and is likely to be allochthonous within mangrove sediments not substantially different from those examined here, in other words, those showing no contributions from sediment horizons that contain charred remains of mangrove trees and root systems. The alternative is to archive samples for future testing. In this way, the sediment’s BC content, and its origin, may be addressed at a later date relatively easily and economically, along with other possible allochthonous recalcitrant organic components, such as kerogens, phytolith–occluded carbon, and possibly microplastics. Finally, we also appeal to researchers involved in terrestrial pyrogenic soil carbon studies to consider the concept of allochthonous recalcitrant carbon when evaluating sequestration services. For example, BC from fossil fuel emissions can be considered an allochthonous recalcitrant when it is produced outside the landscape, along with phytolith–occluded carbon and remobilised kerogens that may be blown or washed in from other landscapes.

## Methods

### Study sites

The sediment cores analysed for black carbon (BC) and total organic carbon (TOC) content were taken from seagrass meadows across a tropical lagoon (Salut–Mengkabong, Malaysia) and a temperate tide-dominated estuary (Little Swanport, Australia). Mangrove forest sediment cores were taken across the tropical system, and data from surface sediments of a subtropical coastal region (Shantou, China) were reanalysed for comparison with the tropical lagoon sediments and the reported results for sediment cores taken from a similar subtropical coastal region in another part of the world (Guanabara Bay, Brazil). The tropical system, Salut–Mengkabong lagoon (6.101734°N, 116.153845°E), is located in a moderately urban setting close to Tuaran, a town to the north of Kota Kinabalu city (population ~100,000); it is situated within the penumbra of the seasonal southern Kalimantan and Java peat fires^23^. We found no charred trees within our mangrove sampling sites, nor was there evidence of recent fires in the Salut–Mengkabong lagoon’s mangrove forests (Supplementary Figure S5), despite intense past El Niño droughts^40^. The temperate system, Little Swanport estuary, Australia (42.3405°S, 147.9380°E), is located in a rural setting and has a history of local forest fires and aboriginal fire management practices^24^. In contrast to the tropical lagoon and temperate estuary, the subtropical mangrove forests of the Shantou wetlands (23.3333°N, 116.7000°E, Shantou, China)^22^ and Guanabara Bay (22.6667°S, 43.3000°W, Rio De Janeiro, Brazil)^19^ are adjacent to highly industralised urban regions with populations of ~5,000,000 and ~12,000,000, respectively. For Guanabara Bay and the Shantou wetlands, the authors assume that the primary source of black carbon in the upper sediments was fossil fuel combustion outside the respective mangrove forest^19,22^. This was supported by data from recent satellite near-real-time observations of fires (Supplementary Figures S6, S7).

### Sampling design

A random hierarchical sampling design encompassing three spatial scales (10^3^, 10^2^, and 10^1^ m) was used in order to accurately estimate carbon contents of sediments in the mangrove forest and seagrass meadows^41,42^ located within the temperate estuary (for seagrass) and tropical lagoon (for seagrass and mangroves) areas (Supplementary Figures S1, S2). In the same manner, a transect hierarchical sampling design was used to obtain a representative estimate between mangrove forests at the estuarine landscape scale^43^ located along the Salut and Mengkabong lagoon branches (over 1 km apart), with transect sampling stations (50 to 100 m long) located every 25 m and running perpendicular to the shoreline (Supplementary Figure S1). All the mangrove sites support an adjacent seagrass meadow. Sediment cores within the Salut–Mengkabong seagrass beds were taken to a depth of 25 cm with a 5-cm-diameter PVC tube (*n* = 57); a hammer Kajak corer (UWITEC™) with a diameter of 6.4 cm was used in Little Swanport (*n* = 24). A surface depth of 25 cm is regarded the minimum required to calculate seagrass carbon stocks^7^. For mangrove sediments, a larger diameter PVC tube (11 cm) was used for easier penetration to 50 cm and to reduce core compaction. Deeper mangrove sediment sections are included in comparison to the seagrass cores to account for the literature’s uncertainty on the depth required to calculate carbon stocks in mangrove forest sediments^6^. All sediment cores were capped immediately after extraction and placed vertically under ice for transport to the laboratory. The cores were then homogenized down to 25 cm, as were additional lower sections (25–30 cm or 25–40 cm) for mangrove cores. The homogenised samples were dried at 60 °C, sieved through a 1-mm mesh, and ground with a porcelain mortar and pestle into fine powder (<63 µm).

### Total organic carbon and black carbon analysis

The dried samples (50 mg) were packed into silver capsules and analysed for TOC content via a LECO macro carbon analyser (model: CHN628), followed by a micro-acidification process according to Gustafsson^25^. The precision of the method, with a coefficient of variation (CV) of ±6.31% (*n* = 14), was assessed across different batches using an in-house batch standard taken from the surface mud of a tropical mangrove forest. We used two different methods, one thermal and one based on chemical oxidation, to isolate the BC fraction before the carbon analysis (as in the TOC procedure, see above). For thermal oxidation, we used a modified version of the standard chemothermal oxidation (CTO) method^25^, thought to isolate BC but with bias to the more refractory charcoal–soot continuum^17^, and concentrated nitric acid oxidation (NAO)^44,45^. The NAO is designed to isolate the more labile macro-chars originating from local fires, which includes other components within the more refractory char–soot continuum^17,45^. Modifications of the CTO method were based on the protocols for larger sample sizes: spreading samples thinly on a single rack of inverted crucible lids at 360 °C^46^ instead of 375 °C, and using a slow temperature ramping rate (30 °C min^−1^)^27^ to inhibit charring combustion within the muffle furnace (Carbolite, Model: CWF 1300). The precision of the CTO method was typically ± 8.99% (*n* = 4), expressed as CV. Modifications to the NAO protocols were restricted to replacing the decanting and washing the sample onto a filter using distilled water, with centrifugation (2,385 g for 10 min) and aspiration. The precision of the method is typically within ±5% standard error^45^.

Samples taken from the Salut–Mengkabong lagoon were collected and analysed in 2016. For Little Swanport estuary, rediscovered remains of archived seagrass sediment samples, collected in 2007, were analysed for BC isolated by CTO in 2014, that is, the samples had been freeze dried, ground, and stored frozen (−25 °C) in the dark. For NAO, all samples collected in 2007 were analysed immediately for black organic matter (BM)^47^ and separately for total organic matter (TOM) using loss on ignition^48^. The results were then expressed as TOC and BC from calibration regressions constructed from seagrass sediments across the sand–mud gradient of the Salut-Mengkabong lagoon (Supplementary Figures S3, S4). It should be noted that the Little Swanport and Salut–Mengkabong protocols for NAO differ in that an attempt was made to extract all the Little Swanport samples from the oxidation tubes. This is in contrast to the Salut-Mengkabong study, in which a weighed subsample was used and corrected for total dry weight remaining after oxidation. The protocol used for the Little Swanport samples may explain some of the additional variability seen in the regression values for BC vs TOC (Fig. 2c, main article). All the BC and TOC data are presented in Supplementary Tables S3, S4, S5 and S6.

### Charring susceptibility during chemothermal oxidation

It has been reported that the CTO method may char existing organic matter, resulting in an overestimation of the BC content within some soil and sediment samples^28^. The source of charring may be either organic components with a high nitrogen content^49^, for example, microalgae^50^, or the low oxygen availability in soil and sediments with high clay content^51^. To test this supposition within the framework of our samples and protocols, a series of standard addition experiments were carried in which microalgal slurries were added to sandy-muddy mangrove sediments.

### Standard addition experiment

#### Methods

Microalgae was sourced as dietary *Spirulina* powder (Organic by Nature, Inc), which was reported as 65% protein, 6% unsaturated fat, 14% carbohydrates, 0.02% sugars, and 0.24% sodium. Standard additions of *Spirulina* were based on the quantity of TOM (as loss on ignition from a 0.45 g dried sample); the additions increased the sediment’s TOM by a factor of around 2 (Supplementary Table S2). The slurry was prepared by mixing the sediment sample with *Spirulina* powder (0.45 g, total dry mass) and distilled water in a 15 mL centrifuge tube, followed by sonication (5 min) to distribute the microalgal contents throughout the sedimentary matrix. The resulting mix was then prepared and combusted according to our modified protocols (see Total organic carbon and black carbon analysis section above), but without an acidification stage, as the results were expressed as BM from loss on ignition at 550 °C.

#### Results and discussion

A Wilcoxon signed-rank test indicated that the median BC content of the mud, muddy sand, and sand mangrove sediments (normalised for additions of *Spirulina*), that is, 2.30%, 2.09%, and 0.59%, respectively, were not statistically significantly different from the median before adding *Spirulina*, that is, 2.58%, 2.23%, and 0.69%, respectively, (*Z* = 1.83, *P* > 0.124) for all three sediment types. Thus, we consider it unlikely that our modified CTO procedures and equipment led to charring of naturally occurring sedimentary organic components; this supposition is supported by the higher BC contents estimated by NAO in the muddy to sandy seagrass beds of Little Swanport estuary and selected seagrass sediments of the Salut–Mengkabong system (see main article).

### Data availability

All data generated or analysed during this study are included in this published article (and its Supplementary Information files).

## Electronic supplementary material

Supplementary information data, figure and table
