# Supplementary material for: Accounting for black carbon lowers estimates of blue carbon storage services
Source: Sci Rep. 2018 Feb 7;8:2553. doi: 10.1038/s41598-018-20644-2 (PMC5803216; doi:10.1038/s41598-018-20644-2)
Supplement: Supplementary file 1 — Supplementary information data, figure and table [file 41598_2018_20644_MOESM1_ESM.pdf]

Accounting for black carbon lowers estimates of blue carbon storage services

Swee Theng Chew[1] and John B. Gallagher[1]

[1]Borneo Marine Research Institute, Universiti Malaysia Sabah, Jalan UMS, 88400 Kota Kinabalu Sabah, Malaysia.

Correspondence and requests for materials should be sent to J. B. Gallagher (john.barry@ums.edu.my)

Data analysis of black carbon variation in spatial scales

A two-way ANOVA was used to statistically test for the black carbon (BC) variation, separately for both CTO and NAO, across difference spatial scales in both seagrass meadows and mangrove forests of Salut-Mengkabong lagoon, and seagrass meadows of Little Swanport estuary. A normality test was performed *a priori* in PAST™ v3.14 statistical tool to make sure data were normally distributed, ($P > 0.05$) before running the parametric test. Any non-normally distributed data was normalized with a two-steps data transformation[1]. The posterior Tukey test, was performed after a two-way ANOVA, to analyses the interaction effect between sites and stations (see Supplementary Table 7).

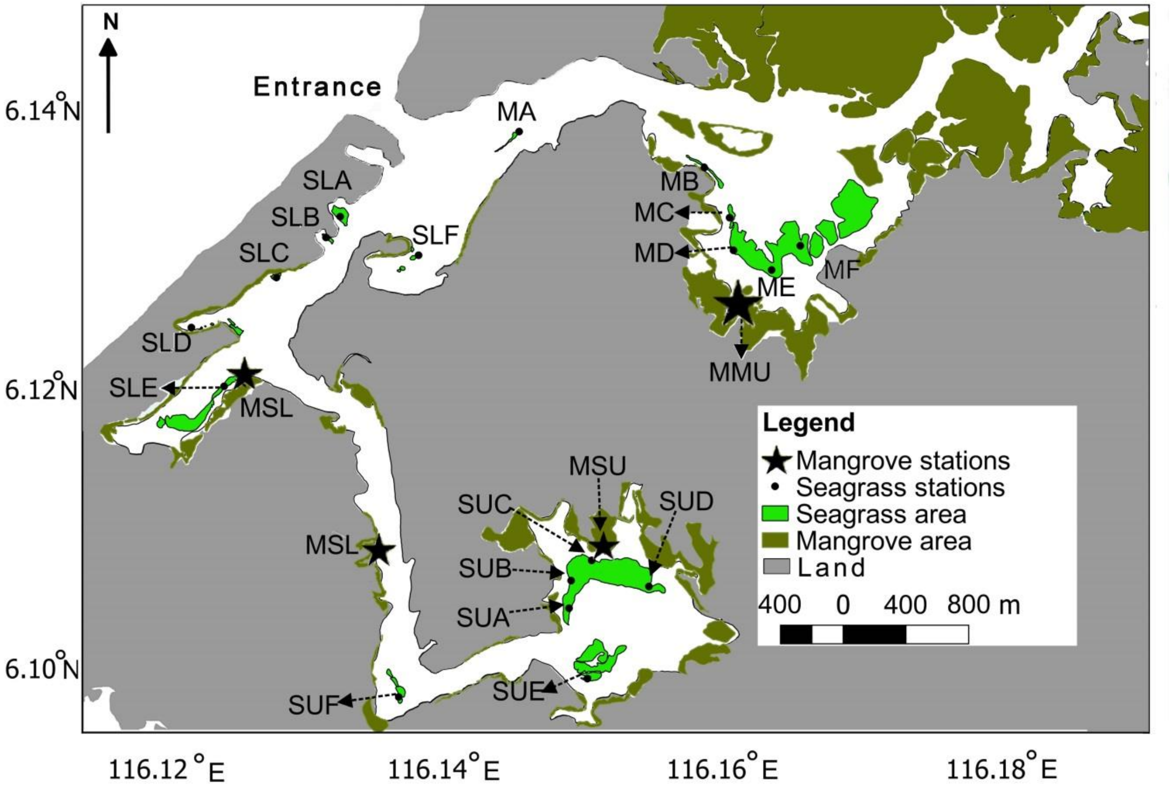

Supplementary Figure S1. Seagrass and mangrove sediment coring stations in Salut-Mengkabong estuary, Malaysia. A hierarchical sampling design with three spatial scales was used for seagrass: regions (upper estuary and lower estuary apart, approximately 10^3 m apart), stations (10^2 m between stations), and plot sites (around 50 m between cores). A total of 18 stations and three plots per station were sampled. For mangrove coring sites, a transect hierarchical sampling design was used, with a total of three plot sites per transect line placed at 25 m intervals. The seagrass and mangrove distribution information were collected based on indigenous knowledge, map data: Google, 2017 DigitalGlobe & TerraMetrics, and ground appraisal by walking and logging GPS seagrass edges at low water springs. The map draw with QGIS v3.18 , http://www.qgis.org/en/site/.

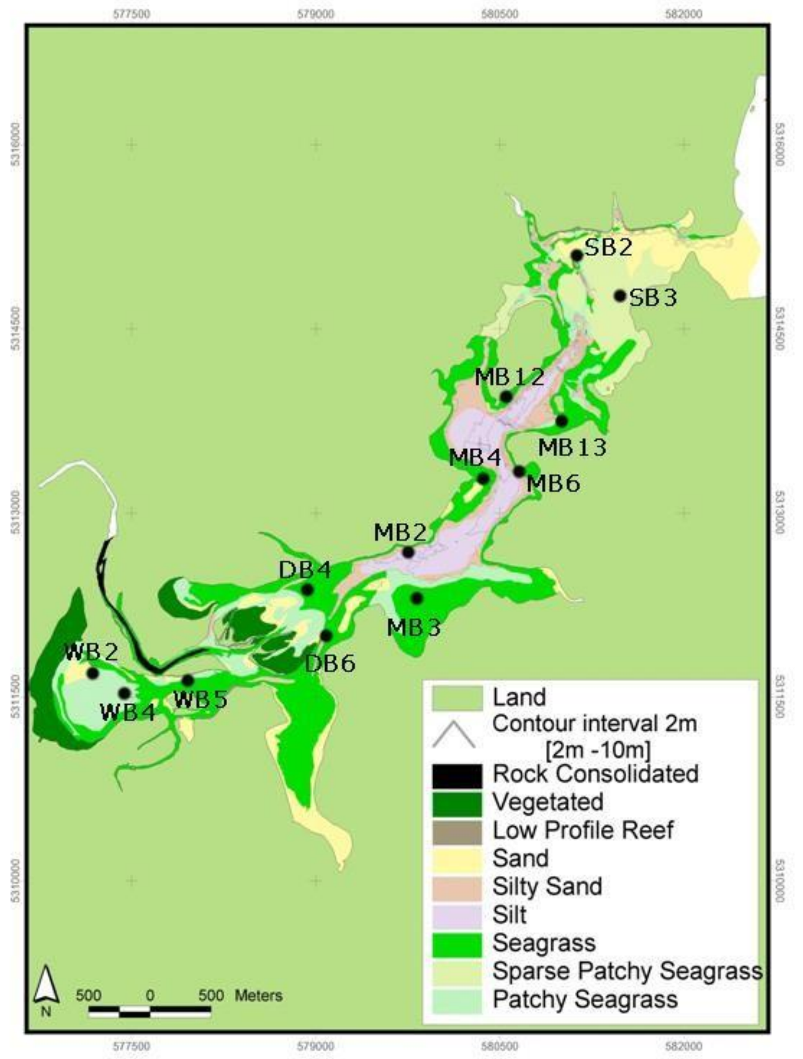

Supplementary Figure S2. The sampling positions of seagrass in Little Swanport estuary, Australia[2]. A hierarchical sampling design was used with three spatial scales: regions (upper estuary, middle estuary, lower estuary, 10^3 m apart), station (10^2 m between stations), and plot site (10's m between cores). "Vegetated area" refers to saltmarsh, located at the head of the estuary

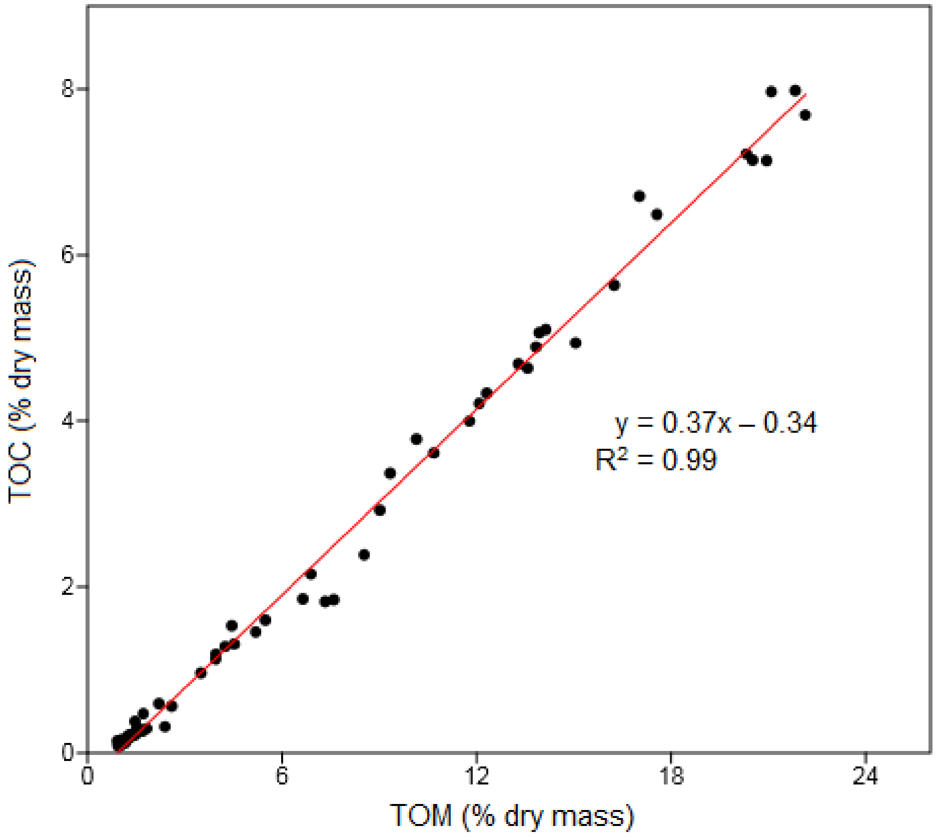

Supplementary Figure S3. Relationship between total organic carbon (TOC) and total organic matter (TOM). There is a strong positive relationship between TOM and TOC (R^2 = 0.99, P < 0.001, n = 55).

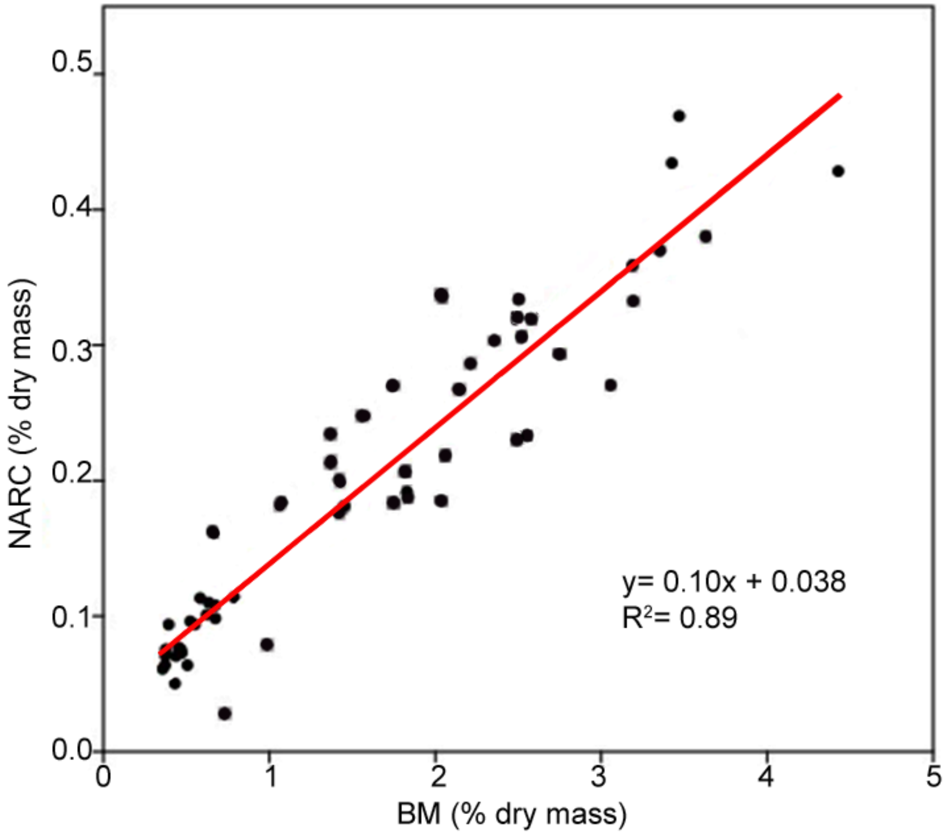

Supplementary Figure S4. Relationship between nitric acid–resistant carbon (NARC), thought to be chars, soots and graphenes as isolated by nitric acid oxidation, and black organic matter (BM). There is a strong positive relationship between NARC and BM (R^2 = 0.89, $P < 0.001$, n = 55).

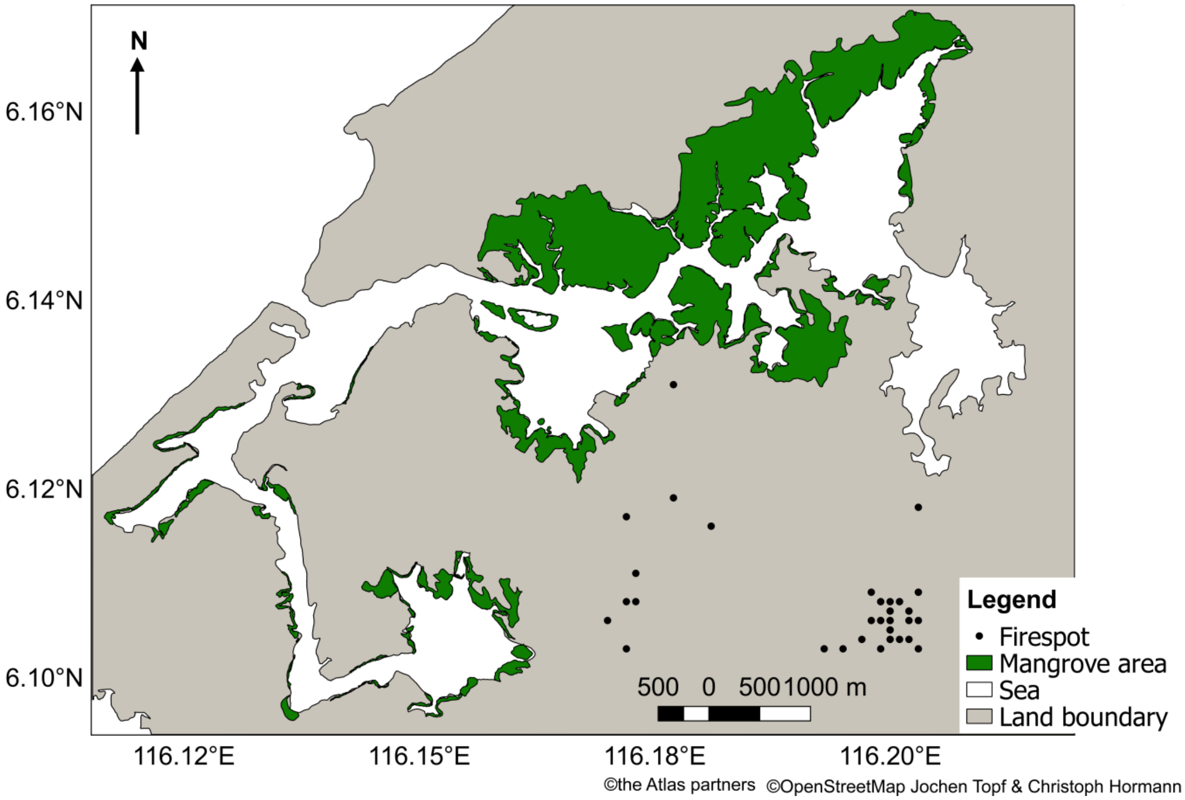

Supplementary Figure S5. A compellation of fires within surrounding Salut–Mengkabong lagoon area (Sabah, Malaysia), as captured by satellite from the year 2000 to 2016. Note all of the fires (●) were situated within into the surrounding hills or nearby settlement, away from the coastal mangrove forests. The mapping tool, QGIS v3.18 used to draw the map with several open source data includes coastal line data from "Open Street Map Data: Coastlines (http://openstreetmapdata.com/data/coastlines)" by Jochen Topf and Christoph Hormann is licensed under CC BY-SA (https://creativecommons.org/licenses/by-sa/2.0/). The mangrove areas data from "World Atlas of Mangrove (version 1.1)" by Mark Spalding, Mami Kainuma & Lorna Collins is licensed under CC BY 4.0. Lastly, fire spot data from NASA fire information for resource management system (FIRMS)[3].

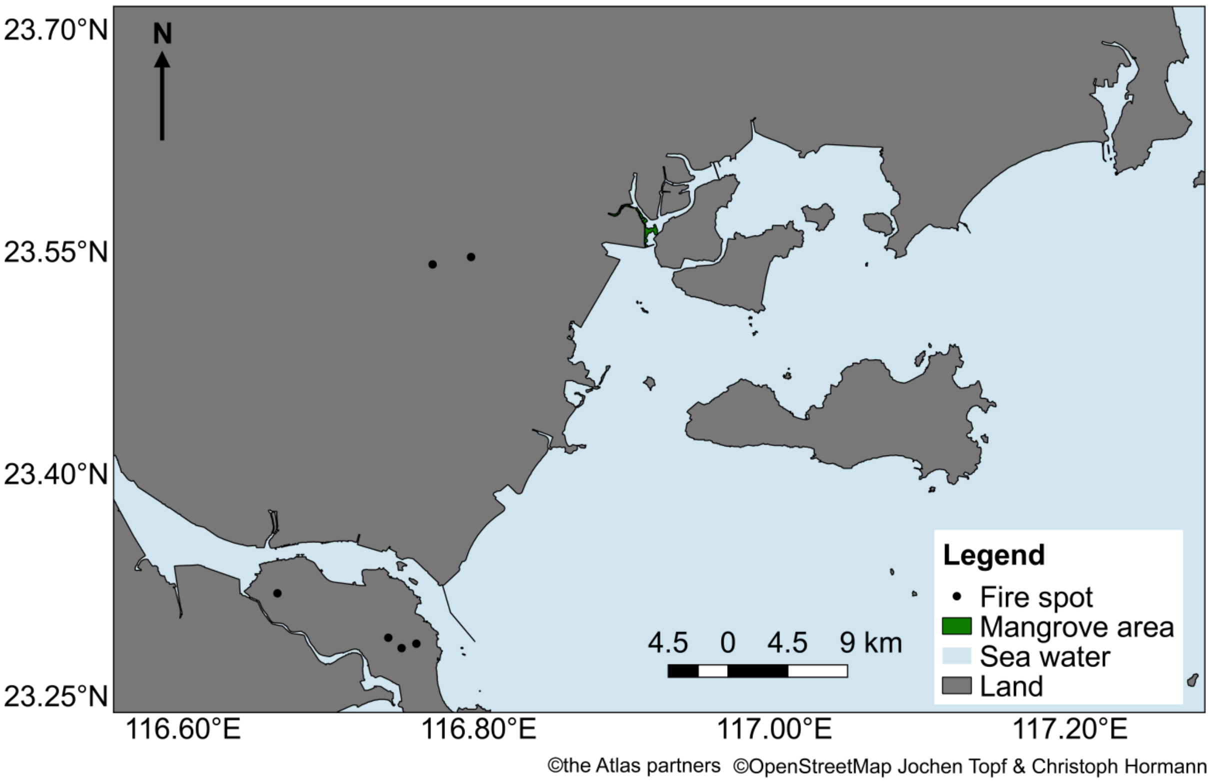

Supplementary Figure S6. A compellation of fires within surrounding Shantou wetland region (China), as captured by satellite from the year 2000 to 2007. Note all of the fires (●) were situated outside of coastal mangrove wetland area. The mapping tool, QGIS v3.18 used to draw the map with several open source data includes coastal line data from "Open Street Map Data: Coastlines (http://openstreetmapdata.com/data/coastlines)" by Jochen Topf and Christoph Hormann is licensed under CC BY-SA (https://creativecommons.org/licenses/by-sa/2.0/). The mangrove areas data from "World Atlas of Mangrove (version 1.1)" by Mark Spalding, Mami Kainuma & Lorna Collins is licensed under CC BY 4.0. Lastly, fire spot data from NASA fire information for resource management system (FIRMS)[3].

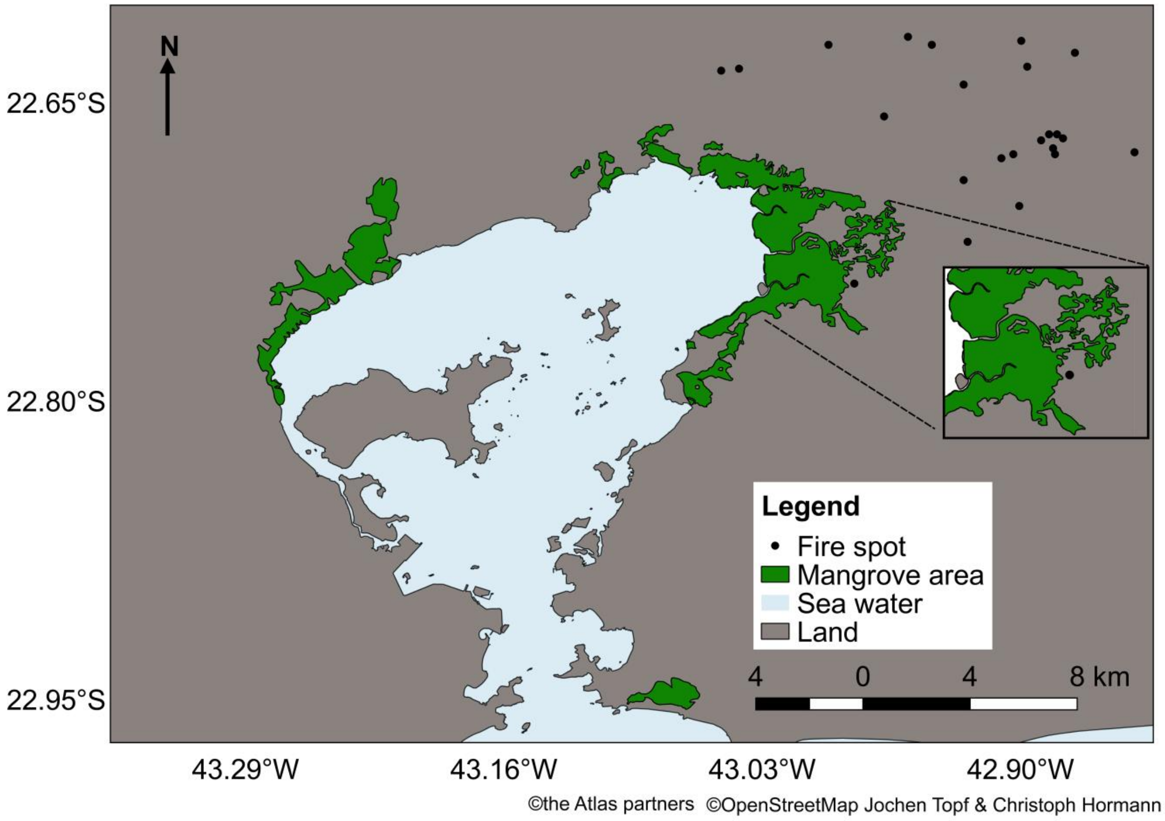

Supplementary Figure S7. A compellation of fires within the surrounding Guanabara Bay area (Brazil), as capture by satellite from the year 2000 to 2004. Note all of the fires (●) were situated outside of the Bay's mangrove forests including the fire spotted close by to the mangrove forest boundary. The mapping tool, QGIS v3.18 used to draw the map with several open source data includes coastal line data from "Open Street Map Data: Coastlines (http://openstreetmapdata.com/data/coastlines)" by Jochen Topf and Christoph Hormann is licensed under CC BY-SA (https://creativecommons.org/licenses/by-sa/2.0/). The mangrove areas data from "World Atlas of Mangrove (version 1.1)" by Mark Spalding, Mami Kainuma & Lorna Collins is licensed under CC BY 4.0. Lastly, fire spot data from NASA fire information for resource management system (FIRMS)[3].

Supplementary Table S1. Linear regression data analysis between total organic carbon (TOC) and Black carbon (BC) with different methods included chemothermal oxidation (CTO), nitric acid oxidation (NAO), potassium dichromate oxidation (PDO) and the Durbin-Watson test (D)[4,5] test for serial correlation across the study areas: Salut-Mengkabong estuary (SM), Little Swanport estuary (LS), and Shantou mangrove demonstration site (S). No serial correlation were detected (*P* = 0.05) in all regression models, with and without intercepts.

No.	Site	BC analysis	Regression equation	Slope s.e.	Intercept s.e.	*R*	*P*	*n*	*D*
a	Seagrass (SM)	CTO	BC = 0.15TOC + 0.01	0.04	0.01	0.97	<0.001	56	1.40
b	Seagrass (SM)	NAO	BC = 0.04TOC + 0.10	0.03	0.01	0.87	<0.001	56	1.50
c	Seagrass (LS)	CTO	BC = 0.06TOC + 0.26	0.01	0.04	0.94	<0.001	14	1.10
d	Seagrass (LS)	NAO	BC = 0.12TOC + 0.22	0.02	0.08	0.78	<0.001	39	1.51
e	Mangrove (SM)	CTO	BC = 0.11TOC + 0.03	0.02	0.07	0.78	<0.001	32	2.25
f	Mangrove (SM)	NAO	BC = 0.01TOC + 0.07	0.01	0.07	0.62	<0.001	32	1.54
g	Mangrove (S)	PDO	BC = 0.05TOC + 0.11	0.03	0.30	0.45	<0.001	14	1.51

Supplementary Table S2. Total organic matter (TOM) and black organic matter (BM) of three sediment samples and their mixture with *Spirulina* (analysed using statistical tool Past™ v3.14).

Samples	TOM (% dry mass)			BM (% dry mass)			Wilcoxon Test	
	Median	25% quartiles	75% quartiles	Median	25% quartiles	75% quartiles	P	Z
Mud	15.10	15.00	15.60	2.58	2.53	2.62	1.83	0.124
Mud and *Spirulina* powder	25.36	25.19	25.66	2.30	2.24	2.39		
Muddy sand	5.06	5.00	5.17	2.22	2.19	2.29	1.83	0.124
Muddy sand and *Spirulina* powder	8.00	7.71	8.13	2.09	1.91	2.10		
Sand	1.13	1.08	1.19	0.69	0.64	0.78	1.83	0.124
Sand and *Spirulina* powder	1.72	1.63	1.92	0.59	0.54	0.61		
Spirulina powder	90.88	90.83	90.91	15.91	14.79	18.81		

References

1. Templeton, G. F. & Templeton, G. F. A Two-Step Approach for Transforming Continuous Variables to Normal : Implications and Recommendations for IS Research. **28,** (2011).
2. Gallagher, J. B. *Natural and Anthropogenic Ecosystem Regime Variance within a Tide-Dominated Estuary: A Late Anthropocene Palaeo-reconstruction* (Ph.D. thesis, University of Tasmania, 2013).
3. Fire Information for Resource Management System (FIRMS). *MODIS Collection 6 NRT Hotspot/Active Fire Detections MCD14DL.* https://earthdata.nasa.gov/firms. DOI: 10.5067/FIRMS/MODIS/MCD14DL.NRT.006 (2017).
4. Savin, N. E. & White, K. J. The Durbin-Watson Test for Serial Correlation with Extreme Sample Sizes or Many Regressors Author (s): N . E . Savin and Kenneth J . White. *Econometrica* **45,** 1989–1996 (1977).
5. Farebrother, R. W. the Durbin-Watson Test for Serial Correlation When There Is No Intercept in the Regression. *Econometrica* **48,** 1553–1563 (1980).

Acknowledgements

We acknowledge the use of data and imagery from LANCE FIRMS operated by the NASA/GSFC/Earth Science Data and Information System (ESDIS) with funding provided by NASA/HQ.

Supplementary Table S3. Little Swanport Estuary's seagrass sediment samples positions, total organic carbon, black carbon contents from CTO, particle size and sediment texture.

**Little Swanport Estuary, Australia (Seagrass)**				
Site	Latitude, Longitude (degrees)	TOC (% dry mass)	BC (% dry mass)	Sediment classification
DB4	42.33684 S 147.95604 E	6.93	0.76	Sandy Mud
MB2A	42.33444 S 147.96694 E	9.00	0.79	Sandy Mud
MB2B	42.33444 S 147.96694 E	9.85	0.76	Sandy Mud
MB2C	42.33444 S 147.96694 E	9.50	0.8	Sandy Mud
MB3A	42.33784 S 147.96886 E	8.42	0.75	Sandy Mud
MB3B	42.33784 S 147.96886 E	10.24	0.78	Sandy Mud
MB3C	42.33784 S 147.96886 E	7.81	0.73	Sandy Mud
WB4A	42.34485 S 147.94174 E	3.76	0.45	Muddy Sand
WB4B	42.34485 S 147.94174 E	1.80	0.24	Muddy Sand
WB4C	42.34485 S 147.94174 E	3.75	0.36	Muddy Sand
SB2A	42.31232 S 147.98296 E	1.63	0.37	Sand
SB3A	42.31593 S 147.98926 E	0.87	0.29	Sand
SB3C	42.31593 S 147.98926 E	2.65	0.51	Sand
SB3B	42.31593 S 147.98926 E	1.94	0.47	Sand

TOC: total organic carbon; BC CTO: soot/ other recalcitrant carbon forms after thermal oxidation (see text for definition delination) .

Supplementary Table S4. Little Swanport Estuary's seagrass sediment samples positions, total organic carbon, black carbon content from NAO, particle size and sediment texture.

**Little Swanport Estuary, Australia (Seagrass)**				
Site	Latitude, Longitude (degrees)	TOC (% dry mass)	NARC (% dry mass)	Sediment classification
WB2A	42.34269 S 147.93684 E	3.41	0.77	Muddy Sand
WB2B	42.34269 S 147.93684 E	2.62	0.60	Muddy Sand
WB2C	42.34269 S 147.93684 E	4.35	0.81	Muddy Sand
WB4A	42.34485 S 147.94174 E	2.12	0.52	Muddy Sand
WB4B	42.34485 S 147.94174 E	2.48	0.82	Muddy Sand
WB4C	42.34485 S 147.94174 E	6.21	1.19	Muddy Sand
WB5A	42.34432 S 147.94698 E	2.22	0.50	Muddy Sand
WB5B	42.34432 S 147.94698 E	1.08	0.26	Muddy Sand
WB5C	42.34432 S 147.94698 E	2.39	0.47	Muddy Sand
DB4A	42.33684 S 147.95604 E	6.35	1.20	Sandy Mud
DB4B	42.33684 S 147.95604 E	6.82	1.28	Sandy Mud
DB4C	42.33684 S 147.95604 E	7.69	0.41	Sandy Mud
DB6A	42.34003 S 147.96059 E	6.77	0.97	Sandy Mud
DB6B	42.34003 S 147.96059 E	7.09	1.43	Sandy Mud
DB6C	42.34003 S 147.96059 E	7.31	1.41	Sandy Mud
MB2A	42.33444 S 147.96694 E	8.02	0.80	Sandy Mud
MB2B	42.33444 S 147.96694 E	8.18	1.39	Sandy Mud
MB2C	42.33444 S 147.96694 E	8.21	1.17	Sandy Mud
MB3A	42.33784 S 147.96886 E	8.21	0.98	Sandy Mud
MB3B	42.33784 S 147.96886 E	8.46	1.04	Sandy Mud
MB3C	42.33784 S 147.96886 E	7.82	1.01	Sandy Mud
MB4A	42.32853 S 147.97510 E	2.72	0.52	Muddy Sand
MB4B	42.32853 S 147.97510 E	3.63	0.88	Muddy Sand
MB4C	42.32853 S 147.97510 E	3.92	0.95	Muddy Sand
MB6A	42.32783 S 147.97934 E	6.01	1.20	Muddy Sand
MB6B	42.32783 S 147.97934 E	6.08	1.10	Muddy Sand
MB6C	42.32783 S 147.97934 E	8.28	1.13	Muddy Sand
MB12A	42.32265 S 147.97789 E	3.60	0.60	Muddy Sand
MB12B	42.32265 S 147.97789 E	3.48	0.55	Muddy Sand
MB12C	42.32265 S 147.97789 E	3.85	0.75	Muddy Sand
MB13A	42.32385 S 147.98268 E	1.06	0.38	Muddy Sand
MB13B	42.32385 S 147.98268 E	4.13	0.87	Muddy Sand
MB13C	42.32385 S 147.98268 E	2.76	0.42	Muddy Sand
SB2A	42.31232 S 147.98296 E	1.67	0.57	Sand
SB2B	42.31232 S 147.98296 E	1.73	0.22	Sand
SB2C	42.31232 S 147.98296 E	2.13	0.17	Sand
SB3A	42.31593 S 147.98926 E	3.73	0.21	Sand
SB3B	42.31593 S 147.98926 E	1.88	0.21	Sand
SB3C	42.31593 S 147.98926 E	2.61	0.21	Sand

TOC: total organic carbon values converted from total organic matter, TOM with equation TOC = 0.37TOM – 0.34.
NARC: nitric acid resistant black carbon values were converted from equation NARC = 0.10BM + 0.038.
(see text for statistic details on conversion equation)

Supplementary Table S5. Salut-Mengkabong Estuary's seagrass sediment samples positions, total organic carbon contents, black carbon contents from two methods, particle size and sediment texture.

Salut-Mengkabong Estuary, Malaysia (Seagrass)								
Site	Latitude, Longitude (degrees)	TOC (% dry mass)	BC CTO (% dry mass)	BC NAO (% dry mass)	Clay (%)	Silt (%)	Sandy (%)	Sediment classification
SUA1	6.10429 N 116.15016 E	4.94	0.73	0.43	19.94	67.23	12.83	Sandy Mud
SUA2	6.10429 N 116.15016 E	1.86	0.30	0.18	9.00	72.49	18.51	Sandy Mud
SUA3	6.10429 N 116.15016 E	4.00	0.55	0.34	15.40	54.86	29.73	Sandy Mud
SUB31	6.10561 N 116.15044 E	4.89	0.70	0.27	1.22	65.58	33.20	Sandy Mud
SUB32	6.10561 N 116.15044 E	7.98	0.97	0.47	1.82	65.89	32.30	Sandy Mud
SUB33	6.10561 N 116.15044 E	7.22	1.15	0.38	15.59	72.75	11.66	Sandy Mud
SUC25	6.10772 N 116.15178 E	4.21	0.87	0.29	5.88	85.24	8.89	Sandy Mud
SUC26	6.10772 N 116.15178 E	4.64	0.82	0.36	6.13	83.33	10.54	Sandy Mud
SUC27	6.10772 N 116.15178 E	7.69	1.16	0.37	9.78	73.43	16.80	Sandy Mud
SUD28	6.10585 N 116.15588 E	7.14	0.97	0.43	0.85	62.90	36.25	Sandy Mud
SUD29	6.10585 N 116.15588 E	7.97	1.24	0.27	5.16	76.47	18.37	Sandy Mud
SUD30	6.10585 N 116.15588 E	7.14	1.21	0.33	0.95	76.09	22.96	Sandy Mud
SUE21	6.09924 N 116.15147 E	1.85	0.30	0.31	2.82	82.65	14.54	Sandy Mud
SUE23	6.09924 N 116.15147 E	1.82	0.28	0.19	23.25	71.80	4.95	Sandy Mud
SUE24	6.09924 N 116.15147 E	2.39	0.34	0.27	24.62	72.83	2.56	Sandy Mud
SUF18	6.09793 N 116.13796 E	1.28	0.20	0.20	15.14	77.26	7.60	Sandy Mud
SUF19	6.09793 N 116.13796 E	1.46	0.26	0.18	15.59	53.45	30.96	Sandy Mud
SUF20	6.09793 N 116.13796 E	0.96	0.22	0.18	20.24	72.97	6.79	Sandy Mud
SLA10	6.13237 N 116.13375 E	0.29	0.02	0.11	14.79	60.22	24.98	Sandy Mud
SLA11	6.13237 N 116.13375 E	0.14	0.02	0.08	6.94	11.11	81.94	Muddy Sand
SLA12	6.13237 N 116.13375 E	0.29	0.04	0.11	3.26	38.97	57.77	Muddy Sand
SLB19	6.13086 N 116.13275 E	0.26	0.04	0.10	48.73	28.88	22.38	Sandy Mud
SLB20	6.13086 N 116.13275 E	0.21	0.03	0.09	29.22	20.53	50.25	Muddy Sand
SLB8	6.13086 N 116.13275 E	0.19	0.03	0.09	17.98	46.03	35.99	Sandy Mud
SLB9	6.13086 N 116.13275 E	0.20	0.03	0.11	17.28	50.93	31.79	Sandy Mud
SLC21	6.12799 N 116.12921 E	0.16	0.02	0.06	1.16	1.62	97.21	Sand
SLC22	6.12799 N 116.12921 E	0.22	0.03	0.07	4.32	10.66	85.02	Muddy Sand
SLC6	6.12799 N 116.12921 E	0.12	0.02	0.06	7.40	31.83	60.77	Muddy Sand
SLD26	6.12440 N 116.12312 E	0.09	0.01	0.07	6.40	7.20	86.40	Muddy Sand
SLD27	6.12440 N 116.12312 E	0.17	0.02	0.06	6.92	17.28	75.80	Muddy Sand
SLD4	6.12440 N 116.12312 E	0.30	0.06	0.08	3.73	27.94	68.34	Muddy Sand
SLD5	6.12440 N 116.12312 E	0.22	0.03	0.10	5.61	33.67	60.72	Muddy Sand
SLE1	6.12019 N 116.12546 E	1.13	0.17	0.18	9.45	83.35	7.20	Sandy Mud
SLE2	6.12019 N 116.12546 E	1.60	0.25	0.21	2.04	81.12	16.84	Sandy Mud
SLE3	6.12019 N 116.12546 E	1.31	0.20	0.21	6.40	83.78	9.82	Sandy Mud
SLF15	6.12959 N 116.13939 E	0.09	0.01	0.05	3.91	29.55	66.54	Muddy Sand
SLF16	6.12959 N 116.13939 E	0.11	0.01	0.07	3.09	66.50	30.41	Sandy Mud
SLF17	6.12959 N 116.13939 E	0.12	0.01	0.08	38.36	19.76	41.88	Sandy Mud
MA40	6.13845 N 116.14659 E	0.56	0.10	0.08	3.89	32.41	63.70	Muddy Sand
MA41	6.13845 N 116.14659 E	0.59	0.11	0.11	10.91	54.48	34.61	Sandy Mud
MA42	6.13845 N 116.14659 E	0.47	0.08	0.16	13.68	62.93	23.39	Sandy Mud
MB43	6.13589 N 116.15983 E	1.19	0.25	0.10	6.03	50.42	43.55	Muddy Sand
MB44	6.13589 N 116.15983 E	0.38	0.08	0.06	6.65	78.68	14.67	Sandy Mud
MB45	6.13589 N 116.15983 E	1.53	0.26	0.03	8.77	72.97	18.26	Sandy Mud
MC46	6.13228 N 116.16166 E	4.69	0.84	0.25	9.40	70.73	19.87	Sandy Mud
MC47	6.13228 N 116.16166 E	6.71	1.19	0.19	14.97	66.77	18.26	Sandy Mud
MC48	6.13228 N 116.16166 E	3.37	0.61	0.23	20.58	61.41	18.26	Sandy Mud
MD49	6.12993 N 116.16194 E	6.49	1.02	0.33	16.59	63.61	19.80	Sandy Mud
MD50	6.12993 N 116.16194 E	5.07	0.89	0.22	5.68	72.48	21.84	Sandy Mud
MD51	6.12993 N 116.16194 E	5.64	0.91	0.23	5.77	14.66	79.58	Muddy Sand
ME52	6.12853 N 116.16467 E	4.33	0.65	0.30	13.16	70.81	16.03	Sandy Mud
ME53	6.12853 N 116.16467 E	5.10	0.76	0.29	17.09	72.62	10.29	Sandy Mud
ME54	6.12853 N 116.16467 E	3.62	0.65	0.32	9.48	81.87	8.65	Sandy Mud
MF55	6.13027 N 116.16672 E	3.78	0.53	0.32	28.77	69.51	1.72	Sandy Mud
MF56	6.13027 N 116.16672 E	2.93	0.45	0.23	25.49	68.43	6.08	Sandy Mud
MF57	6.13027 N 116.16672 E	2.16	0.36	0.19	16.41	74.70	8.89	Sandy Mud
MTBS (bare sed.)	6.142841 N 116.150380 E	0.32	0.07	0.09	7.21	33.18	59.61	Muddy Sand

TOC: total organic carbon; BC CTO: soot/ other recalcitrant carbon forms after thermal oxidation; BC NAO: char- soot carbon after nitric acid digestion (see text for definition delination) .
MTBS: sample from bare sediment to compare with samples from seagrass bed.

Supplementary Table S6. Salut-Mengkabong Estuary's mangrove sediment samples positions, total organic carbon contents, black carbon contents from two methods, particle size and sediment texture.

Salut-Mengkabong Estuary, Malaysia (Mangrove)									
Site/ Sample	Latitude, Longitude (degrees)	Depth in Sediment (cm)	TOC (% dry mass)	BC CTO (% dry mass)	BC NAO (% dry mass)	Clay (%)	Silt (%)	Sandy (%)	Sediment Classification
MSU1.0	6.10854N 116.15240 E	0 - 25	6.29	0.66	0.21	2.79	20.50	76.71	Muddy Sand
MSU1.0	6.10854N 116.15240 E	25 - 40	7.51	0.74	0.23	3.76	27.88	68.35	Muddy Sand
MSU1.1	6.10854N 116.15240 E	0 - 25	5.83	0.72	0.09	4.67	24.72	70.61	Muddy Sand
MSU1.1	6.10854N 116.15240 E	25 - 44	6.00	0.73	0.14	4.16	34.90	60.94	Muddy Sand
MSU2.0	6.10854N 116.15240 E	0 - 23	2.69	0.48	0.07	2.58	24.83	72.60	Muddy Sand
MSU2.1	6.10854N 116.15240 E	0 - 22	5.79	0.88	0.09	2.03	34.04	63.93	Muddy Sand
MSU3.0	6.10854N 116.15240 E	0 - 25	5.25	0.82	0.09	4.58	28.15	67.28	Muddy Sand
MSU3.0	6.10854N 116.15240 E	25-34	7.90	0.12	0.14	5.57	25.02	69.41	Muddy Sand
MSU3.1	6.10854N 116.15240 E	0 - 25	7.10	1.39	0.10	3.04	34.53	62.43	Muddy Sand
MSU3.1	6.10854N 116.15240 E	25 - 31	6.48	1.36	0.14	1.98	27.73	70.29	Muddy Sand
MSL4.0	6.10855 N 116.13644 E	0 - 25	0.76	0.12	0.06	4.76	61.08	34.16	Sandy Mud
MSL4.1	6.10855 N 116.13644 E	25 - 42	2.08	0.20	0.12	3.70	40.31	55.99	Muddy Sand
MSL4.2	6.10855 N 116.13644 E	0 - 25	0.95	0.14	0.05	4.55	54.27	41.18	Sandy Mud
MSL4.2	6.10855 N 116.13644 E	25-30	1.45	0.10	0.07	4.47	60.53	35.00	Sandy Mud
MSL5.0	6.12054 N 116.12672 E	0 - 25	0.83	0.13	0.08	5.98	58.07	35.95	Sandy Mud
MSL5.0	6.12054 N 116.12672 E	25 - 43	1.83	0.26	0.09	5.05	37.65	57.30	Muddy Sand
MSL5.1	6.12054 N 116.12672 E	0 - 25	1.02	0.15	0.08	5.30	74.78	19.92	Sandy Mud
MSL5.1	6.12054 N 116.12672 E	25 - 38	1.86	0.25	0.06	3.58	53.80	42.62	Muddy Sand
MSL9.0	6.12054 N 116.12672 E	0-25	1.81	0.18	0.13	8.67	81.26	10.06	Sandy Mud
MSL9.0	6.12054 N 116.12672 E	25-28	1.77	0.16	0.10	4.69	79.52	15.79	Sandy Mud
MSL9.1	6.12054 N 116.12672 E	0-25	1.09	0.13	0.17	7.81	69.81	22.37	Sandy Mud
MSL9.1	6.12054 N 116.12672 E	25-31	4.44	0.41	0.13	3.42	37.73	58.85	Muddy Sand
MSL10.0	6.12054 N 116.12672 E	0-27	1.55	0.26	0.07	3.94	31.57	64.49	Muddy Sand
MSL10.1	6.12054 N 116.12672 E	0-23	2.07	0.33	0.04	2.20	22.27	75.53	Muddy Sand
MMU 6.0	6.12609 N 116.16269 E	0 - 28	7.52	0.81	0.19	4.32	40.62	55.06	Muddy Sand
MMU 6.1	6.12609 N 116.16269 E	0 - 28	7.54	0.85	0.19	3.81	46.88	49.31	Muddy Sand
MMU 7.0	6.12609 N 116.16269 E	0 - 25	5.44	0.77	0.14	5.05	45.48	49.47	Muddy Sand
MMU 7.1	6.12609 N 116.16269 E	0 - 25	4.19	0.49	0.08	4.26	22.73	73.01	Muddy Sand
MMU 8.0	6.12609 N 116.16269 E	0 - 25	3.15	0.35	0.14	2.94	60.06	37.00	Sandy Mud
MMU 8.0	6.12609 N 116.16269 E	25 - 31	1.36	0.13	0.12	4.65	49.49	45.86	Muddy Sand
MMU 8.1	6.12609 N 116.16269 E	0 - 25	4.09	0.46	0.11	4.20	73.76	22.04	Sandy Mud
MMU 8.1	6.12609 N 116.16269 E	25 - 30	3.94	0.38	0.10	4.25	66.09	29.65	Sandy Mud

TOC: total organic carbon; BC CTO: soot/ other recalcitrant carbon forms after thermal oxidation; BC NAO: char- soot carbon after nitric acid digestion (see text for definition delination) .

Supplementary Table S7.1. The table shows the variation of black carbon content isolated by nitric acid oxidation (BC NAO) between Salut-Mengkabong lagoon's mangrove. The analysis demonstartes that most of the variance is at the site scale (~1km).

a. The comparison of BC NAO among sites and stations at Salut-Mengkabong lagoon's mangrove with two-way ANOVA.

	Sum of sqrs	df	Mean square	F	*P* (same)
sites:	0.14	2	0.07	5.09	**0.03**
stations:	0.10	2	0.05	3.56	0.07
Interaction:	0.22	4	0.06	4.16	**0.04**
Within:	0.12	9	0.01		
Total:	0.58	17			

b. The comparison of BC NAO among sites at Salut-Mengkabong lagoon's mangrove.

	SU	M	SL
SU		0.64	0.13
M	0.64		**0.03**
SL	0.13	**0.03**	

SU: Salut upper lagoon; M: Mengkabong; SL: Salut lower lagoon

c. Interaction effect between Salut-Mengkabong lagoon's mangrove sites and stations analysed with Turkey's post-hoc test.

		P value
SU-a	SU-b	0.25
SU-a	SU-c	0.89
SU-a	M-a	1.00
SU-a	SL-a	0.24
SU-b	SU-c	0.80
SU-b	M-b	0.94
SU-b	SL-b	0.57
SU-c	M-c	1.00
SU-c	SL-c	0.13
M-a	M-b	0.41
M-a	M-c	0.59
M-a	SL-a	0.11
M-b	M-c	1.00
M-b	SL-b	0.98
M-c	SL-c	0.13
SL-a	SL-b	0.56
SL-a	SL-c	0.67
SL-b	SL-c	0.07

a,b,c: stations along the same transect line, 25 m apart

Supplementary Table S7.2. The table shows the variation of black carbon content, as isolated by chemothermal oxidation (BC CTO), between Salut-Mengkabong lagoon's mangroves. The analysis indicates differences were at the site scale (~1 km) along the individual Salut and Mengkabong branches of the lagoon(Figure S1).

a. The comparison of BC CTO among sites and stations at Salut-Mengkabong lagoon's mangrove with two-way ANOVA.

	Sum of sqrs	df	Mean square	F	*P* (same)
sites:	0.79	2	0.40	16.46	**9.85E-04**
stations:	0.01	2	0.01	0.28	0.76
Interaction:	0.21	4	0.05	2.22	0.15
Within:	0.22	9	0.02		
Total:	1.24	17			

b. The comparison of BC CTO among sites at Salut-Mengkabong lagoon's mangrove.

	SU	M	SL
SU		0.41	**1.13E-03**
M	0.41		**6.39E-03**
SL	**1.13E-03**	**6.39E-03**	

SU: Salut upper lagoon; M: Mengkabong; SL: Salut lower lagoon

c. Interaction effect between Salut-Mengkabong lagoon's mangrove sites and stations analysed with Turkey's post-hoc test.

		P value
SU-a	SU-b	1.00
SU-a	SU-c	1.00
SU-a	M-a	1.00
SU-a	SL-a	0.06
SU-b	SU-c	0.99
SU-b	M-b	1.00
SU-b	SL-b	0.12
SU-c	M-c	0.23
SU-c	SL-c	0.17
M-a	M-b	0.97
M-a	M-c	0.20
M-a	SL-a	**0.03**
M-b	M-c	0.55
M-b	SL-b	0.14
M-c	SL-c	1.00
SL-a	SL-b	1.00
SL-a	SL-c	0.91
SL-b	SL-c	0.96
SL-b	SL-c	0.96

a,b,c: stations along the same transect line, 25 m apart

Supplementary Table S7.3. The table shows the variation of black carbon content, as isolated by nitric acid oxidation (BC NAO), between Salut-Mengkabong lagoon's seagrass meadows. The analysis indicates there were significant differences among all sites. At the station scale, differences were found between SU-F and SU-B within Salut, and stations M-B were found to be different with station M-D, M-E and M-F more sheltered area within Mengkabong.

a. The comparison of BC NAO among sites and stations at Salut-Mengkabong lagoon's seagrass meadows with two-way ANOVA.

	Sum of sqrs	df	Mean square	F	*P* (same)
sites:	26.37	2	13.19	37.51	**1.57E-09**
stations:	2.99	5	0.60	1.70	0.16
Interaction:	18.02	10	1.80	5.13	**1.21E-04**
Within:	12.65	36	0.35		
Total:	60.04	53			

b. The comparison of BC NAO among sites at Salut-Mengkabong lagoon's seagrass meadows.

	SU	M	SL
SU		**2.33E-04**	**1.27E-04**
M	**2.33E-04**		**1.02E-03**
SL	**1.27E-04**	**1.02E-03**	

SU: Salut upper lagoon; M: Mengkabong; SL: Salut lower lagoon

c. Interaction effect between Salut-Mengkabong lagoon's seagrass meadows sites and stations analysed with Turkey's post-hoc test.

		P value
SU-A	SU-B	0.66
SU-A	SU-C	1.00
SU-A	SU-D	1.00
SU-A	SU-E	1.00
SU-A	SU-F	0.60
SU-A	M-A	0.18
SU-A	SL-A	0.12
SU-B	SU-C	0.88
SU-B	SU-D	0.92
SU-B	SU-E	0.15
SU-B	SU-F	**8.32E-03**
SU-B	M-B	**1.28E-04**
SU-B	SL-B	**5.23E-04**
SU-C	SU-D	1.00
SU-C	SU-E	0.96
SU-C	SU-F	0.34
SU-C	M-C	0.79
SU-C	SL-C	**7.46E-04**
SU-D	SU-E	0.94
SU-D	SU-F	0.29
SU-D	M-D	0.97
SU-D	SL-D	**3.31E-03**
SU-E	SU-F	0.99
SU-E	M-E	1.00
SU-E	SL-E	1.00
SU-F	M-F	1.00
SU-F	SL-F	0.46
M-A	M-B	0.71
M-A	M-C	0.93
M-A	M-D	0.62
M-A	M-E	0.33
M-A	M-F	0.85
M-A	SL-A	1.00
M-B	M-C	0.05
M-B	M-D	**0.01**
M-B	M-E	**3.12E-03**
M-B	M-F	**0.03**
M-B	SL-B	0.85
M-C	M-D	1.00
M-C	M-E	0.99
M-C	M-F	1.00
M-C	SL-C	0.09
M-D	M-E	1.00
M-D	M-F	1.00
M-D	SL-D	0.10
M-E	M-F	1.00
M-E	SL-E	0.92
M-F	SL-F	0.08
SL-A	SL-B	1.00
SL-A	SL-C	0.91
SL-A	SL-D	1.00
SL-A	SL-E	0.98
SL-A	SL-F	0.96
SL-B	SL-C	0.93
SL-B	SL-D	1.00
SL-B	SL-E	0.98
SL-B	SL-F	0.97
SL-C	SL-D	1.00
SL-C	SL-E	0.23
SL-C	SL-F	1.00
SL-D	SL-E	0.60
SL-D	SL-F	1.00
SL-E	SL-F	0.31

A, B, C, D, E & F: stations (apart ~ 100s' m)

Supplementary Table S7.4. The table shows the variation of black carbon content isolated by chemothermal (BC CTO) between Salut-Mengkabong lagoon's seagrass meadows. The analysis indicates significant differences among all sites. Over station scales, station SU-D in Salut upper lagoon BC CTO, was significantly different to the remaining stations in the same site. In Mengkabong, both stations, M-A and M-B, were significantly different to station M-C, M-D and station SL-E and SL-F in Salut lower lagoon.

a. The comparison of BC CTO among sites and stations at Salut-Mengkabong lagoon's seagrass meadows with two-way ANOVA.

	Sum of sqrs	df	Mean square	F	*P* (same)
sites:	31.73	2	15.87	88.86	**1.19E-14**
station:	10.43	5	2.09	11.69	**9.38E-07**
Interaction:	11.44	10	1.14	6.407	**1.41E-05**
Within:	6.43	36	0.18		
Total:	60.04	53			

b. The comparison of BC CTO among sites at Salut-Mengkabong lagoon's seagrass meadows.

	SU	M	SL
SU		**0.01**	**1.27E-04**
M	**0.01**		**1.27E-04**
SL	**1.27E-04**	**1.27E-04**	

SU: Salut upper lagoon; M: Mengkabong; SL: Salut lower lagoon

c. Interaction effect between Salut-Mengkabong lagoon's seagrass meadows sites and stations analysed with Turkey's post-hoc test.

		P value
SU-A	SU-B	0.73
SU-A	SU-C	0.68
SU-A	SU-D	**2.19E-04**
SU-A	SU-E	1.00
SU-A	SU-F	0.92
SU-A	M-A	0.45
SU-A	SL-A	**0.01**
SU-B	SU-C	1.00
SU-B	SU-D	**0.03**
SU-B	SU-E	0.25
SU-B	SU-F	0.05
SU-B	M-B	**0.03**
SU-B	SL-B	**4.30E-04**
SU-C	SU-D	**0.03**
SU-C	SU-E	0.21
SU-C	SU-F	**0.04**
SU-C	M-C	1.00
SU-C	SL-C	**1.41E-04**
SU-D	SU-E	**1.32E-04**
SU-D	SU-F	**1.27E-04**
SU-D	M-D	**0.04**
SU-D	SL-D	**1.27E-04**
SU-E	SU-F	1.00
SU-E	M-E	0.97
SU-E	SL-E	1.00
SU-F	M-F	0.98
SU-F	SL-F	**1.79E-03**
M-A	M-B	1.00
M-A	M-C	**8.18E-03**
M-A	M-D	**3.92E-03**
M-A	M-E	0.18
M-A	M-F	0.65
M-A	SL-A	0.88
M-B	M-C	**0.04**
M-B	M-D	**0.02**
M-B	M-E	0.48
M-B	M-F	0.94
M-B	SL-B	0.91
M-C	M-D	1.00
M-C	M-E	0.98
M-C	M-F	0.60
M-C	SL-C	**1.54E-04**
M-D	M-E	0.91
M-D	M-F	0.43
M-D	SL-D	**1.36E-04**
M-E	M-F	1.00
M-E	SL-E	0.44
M-F	SL-F	**1.60E-04**
SL-A	SL-B	1.00
SL-A	SL-C	1.00
SL-A	SL-D	1.00
SL-A	SL-E	0.58
SL-A	SL-F	0.52
SL-B	SL-C	1.00
SL-B	SL-D	1.00
SL-B	SL-E	0.93
SL-B	SL-F	0.18
SL-C	SL-D	1.00
SL-C	SL-E	0.46
SL-C	SL-F	0.64
SL-D	SL-E	0.44
SL-D	SL-F	0.67
SL-E	SL-F	**4.35E-03**

A, B, C, D, E & F: stations (apart ~ 100s' m)

Supplementary Table S7.5. The table shows the variation of black carbon content isolated by chemothermal (BC CTO) between Little Swanport estuary's seagrass meadows. Overall, no significant different were found across different spatial scales.

a. The comparison of BC CTO among sites and stations at Little Swanport estuary's seagrass meadows with two-way ANOVA.

	Sum of sqrs	df	Mean square	F	*P* (same)
sites:	10.44	2	5.22	2.35	0.24
stations:	1.23	2	0.62	0.28	0.78
Interaction:	1.41	4	0.35	0.16	0.95
Within:	6.66	3	2.22		
Total:	19.73	11			

b. The comparison of BC CTO among sites at Little Swanport estuary's seagrass meadows.

	LSM	LSU	LSL
LSM		0.26	0.38
LSU	0.26		0.93
LSL	0.38	0.93	

LSM: Little Swanport middle estuary refers to area where stations MB was located (see Supplementary Figure S2)
LSU: Little Swanport upper estuary refers to area where station WB were located (see Supplementary Figure S2);
LSL: Littile Swanport lower estuary refers to area where station SB was located (see Supplementary Figure S2)

c. Interaction effect between Little Swanport estuary's seagrass meadows sites and stations analysed with Turkey's post-hoc test.

		P value
LSM-a	LSM-b	1.00
LSM-a	LSM-c	0.98
LSM-a	LSU-a	0.98
LSM-a	LSL-a	0.91
LSM-b	LSM-c	0.97
LSM-b	LSU-b	0.85
LSM-b	LSL-b	1.00
LSM-c	LSU-c	0.75
LSM-c	LSL-c	0.85
LSU-a	LSU-b	1.00
LSU-a	LSU-c	1.00
LSU-a	LSL-a	1.00
LSU-b	LSU-c	1.00
LSU-b	LSL-b	0.99
LSU-c	LSL-c	1.00
LSL-a	LSL-b	1.00
LSL-a	LSL-c	1.00
LSL-b	LSL-c	1.00

a, b, c: stations (100's m apart)

Supplementary Table S7.6. The table shows the variation of black carbon content isolated by nitric acid (BC NAO) between Little Swanport estuary's seagrass meadows. Overall, there were significant differences of BC NAO among all sites.

a. The comparison of BC NAO among sites and stations at Little Swanport estuary's seagrass meadows with two-way ANOVA.

	Sum of sqrs	df	Mean square	F	*P* (same)
sites:	22.23	3	7.41	11.51	**4.86E-05**
stations:	1.43	2	0.72	1.11	0.34
Interaction:	4.24	6	0.71	1.10	0.39
Within:	17.38	27	0.64		
Total:	45.28	38			

b. The comparison of BC NAO among sites at Little Swanport estuary's seagrass meadows.

	LSU	LSMU	LSML	LSL
LSU		**0.01**	0.89	0.07
LSMU	**0.01**		**0.04**	**1.77E-04**
LSML	0.89	**0.04**		**9.29E-03**
LSL	0.07	**1.77E-04**	**9.29E-03**	

LSU: Little Swanport upper estuary refers to area where stations WB was located (see Supplementary Figure 2)
LSMU: The middle part of Little Swanport estuary, closer to upper estuary, where station DB and MB2 & 3 were located (see Supplementary Figure S2)
LSML: The middle part of Little Swanport estuary, closer to lower estuary, where stations MB 4,6,12 & 13 were located (see Supplementary Figure S2)
LSL: Littile Swanport lower estuary refers to area where station SB was located (see Supplementary Figure S2)

c. Interaction effect between Little Swanport estuary's seagrass meadows sites and stations analysed with Turkey's post-hoc test.

		P value
LSU-a	LSU-b	1.00
LSU-a	LSU-c	0.99
LSU-a	LSMU-a	0.81
LSU-a	LSML-a	1.00
LSU-a	LSL-a	1.00
LSU-b	LSU-c	0.99
LSU-b	LSMU-b	**0.02**
LSU-b	LSML-b	0.98
LSU-b	LSL-b	0.90
LSU-c	LSMU-c	0.99
LSU-c	LSML-c	1.00
LSU-c	LSL-c	0.22
LSMU-a	LSMU-b	0.32
LSMU-a	LSMU-c	1.00
LSMU-a	LSML-a	0.95
LSMU-a	LSL-a	0.45
LSMU-b	LSMU-c	0.43
LSMU-b	LSML-b	0.12
LSMU-b	LSL-b	**1.86E-03**
LSMU-c	LSML-c	0.97
LSMU-c	LSL-c	**0.03**
LSML-a	LSML-b	1.00
LSML-a	LSML-c	1.00
LSML-a	LSL-a	0.94
LSML-b	LSML-c	1.00
LSML-b	LSL-b	0.37
LSML-c	LSL-c	0.19
LSL-a	LSL-b	1.00
LSL-a	LSL-c	0.96
LSL-b	LSL-c	1.00

a, b, c: stations (100's m apart)
